# Agouti Signaling Protein and Its Receptors as Potential Molecular Markers for Intramuscular and Body Fat Deposition in Cattle

**DOI:** 10.3389/fphys.2018.00172

**Published:** 2018-03-06

**Authors:** Yinuo Liu, Elke Albrecht, Lisa Schering, Christa Kuehn, Runjun Yang, Zhihui Zhao, Steffen Maak

**Affiliations:** ^1^College of Animal Science, Jilin University, Changchun, China; ^2^Institute of Muscle Biology and Growth, Leibniz Institute for Farm Animal Biology, Dummerstorf, Germany; ^3^Institute for Genome Biology, Leibniz Institute for Farm Animal Biology, Dummerstorf, Germany

**Keywords:** adipokine, agouti signaling protein, melanocortin receptor, attractin, bovine, body composition, fat deposition

## Abstract

Transcriptome analyses of bovine muscle tissue differing in intramuscular fat (IMF) content identified agouti signaling protein (ASIP) as a promising candidate gene for fat deposition. The protein is secreted from adipocytes and may serve as a signaling molecule in cross-talk between adipocytes and muscle fibers or other cells. Known receptors for ASIP are the melanocortin receptors (e.g., MC4R) and attractin (ATRN). The present study was conducted to determine relationships between the expression of *ASIP* and its receptors in different bovine tissues with fat deposition. Adipose tissues, liver, and longissimus muscle tissue were collected from 246 F_2_-generation bulls (Charolais × Holstein cross) and gene expression was measured with RT-qPCR. During analysis of subcutaneous fat (SCF) of all bulls, 17 animals were identified with a transposon-derived transcript (Exon2C) inserted in the *ASIP* gene and dramatically increased *ASIP* mRNA levels. Significant correlations between normalized mRNA values of SCF and phenotypic traits related to fat deposition were found in bulls without Exon2C. Three retrospectively assigned groups [Exon2C, *n* = 17; high carcass fat (HCF), *n* = 20; low carcass fat (LCF), *n* = 20] were further analyzed to verify expression differences and elucidate molecular reasons. Expression of *ASIP* could be detected in isolated muscle fibers and adipocytes of Exon2C bulls in contrast to HCF and LCF bulls, indicating ectopic *ASIP* expression if the transposon is present. Among adipose tissues, highest *ASIP* mRNA levels were measured in SCF with significantly higher values in HCF compared to LCF bulls (1.6-fold, *P* < 0.05). However, the protein abundance was below the detection limit in all bulls. Potential ASIP receptors were detected in most investigated tissues. The expression of *MC4R* was higher and of *ATRN* was lower in several tissues of LCF compared to HCF bulls, whereas *MC1R* was not differentially expressed. Bulls of the Exon2C group had lower *ATRN* mRNA values than HCF and LCF bulls in perirenal fat (PF), but higher (*P* < 0.05) values in muscle. Receptors were also expressed in tissues where *ASIP* mRNA was not detected. Consequently, those tissues could be targets for ASIP if it circulates.

## Introduction

Understanding molecular mechanisms that control body composition and meat quality is important to improve efficiency and sustainability of beef production. Muscle and fat are competitors for nutrients. Thus, the conversion of feed to muscle and fat determines the efficiency of meat production (Sillence, 2004). Different factors, such as breed, age, gender, and feed composition, but also circulating factors like hormones, are known to influence accretion of nutritional energy in the form of muscle or fat (Hocquette, 2010). Signaling molecules may be involved in the determination whether nutrients are converted to muscle mass or stored as fat and in recruitment of new cells for the adipose lineage. The current study focused on a particular adipokine the agouti signaling protein (ASIP) which has been associated with obesity and insulin resistance in studies in mice and humans (Yen et al., 1994; Smith et al., 2003). Former investigations of *ASIP* in cattle have mainly focused on coat color (Girardot et al., 2006). Since *ASIP* mRNA could also be detected in different tissues, including adipose tissue, a role outside melanogenesis was suggested (Sumida et al., 2004). Girardot et al. (2006) described a full-length long interspersed nuclear element (L1-BT, Exon2C) inserted in the *ASIP* gene which promotes overexpression of *ASIP* mRNA. This Exon2C was responsible for detection of *ASIP* as top candidate for intramuscular fat (IMF) deposition in a comparison of muscle transcriptomes of Japanese Black and Holstein steers (Albrecht et al., 2012). It is not clear whether this overexpression has an effect on fat deposition, but both breeds developed clearly different amounts of IMF under the same high energy feeding conditions established in Japan to maximize marbling (Albrecht et al., 2011b). The study of Sadkowski et al. (2014) identified also *ASIP* as top candidate for differential expressed genes in a three breed comparison with differences in IMF deposition. It can only be speculated that Exon2C was responsible for the large expression difference observed between Hereford and Limousin. Nevertheless, a recent study supports associations between *ASIP* and nutrient accretion in cattle (Kern et al., 2016). It was one of the highest regulated genes when comparing cattle differing in feed intake and daily gain, important parameters for efficiency of beef production. The expression of *ASIP* can possibly be influenced by feeding. A study in pigs showed increased *ASIP* expression in the group receiving a high-fat-diet (Zhao et al., 2015). Modulation of gene expression by nutritional intervention is the most acceptable way to control processes involved in body or muscle composition.

A prerequisite for the function of ASIP as a signaling molecule, with auto-, para- or endocrine effects, is the expression of respective receptors. Ollmann et al. (1998) have demonstrated that ASIP binds to the melanocortin receptor 1 (MC1R) competing with α-melanocyte stimulating hormone (α-MSH) influencing pigment production in melanocytes. Further members of the melanocortin receptor family (MC2R, MC4R) can also be targeted by ASIP (Yang et al., 1997). The MC4R is mainly expressed in hypothalamus but also in adipose tissue and is involved in appetite regulation (Adan et al., 2006). In cattle, a single nucleotide polymorphism was found in the *MC4R* gene strongly associated with backfat thickness and marbling (Liu et al., 2010). Polymorphisms in different melanocortin receptors were associated with adipose tissue accumulation, feed conversion and daily gain, thus influencing body composition in different species (reviewed by Switonski et al., 2013). Another receptor for ASIP with lower affinity was described by He et al. (2001), namely attractin (Atrn). Their study with transgenic mice suggested that interaction between Atrn and agouti is necessary for signaling through Mc1r and probably also through Mc4r. Furthermore, they demonstrated that the absence of Atrn suppressed agouti induced obesity. Associations between the *ATRN* gene and body weight and fatness in pigs were demonstrated by Kim et al. (2005).

The aim of the present study was to determine associations between gene expression of *ASIP* and its receptors in different tissues with fat deposition in bulls with similar genetic background and identical housing and feeding conditions. Bulls in this study originated from a F_2_-generation of an experimental Charolais × Holstein cross and varied in IMF content and body composition. Furthermore, potential auto- and paracrine effects of ASIP on bovine tissues were considered.

## Materials and methods

### Animals and sampling

Crossbred bulls (*n* = 246, F_2_-generation, Charolais × Holstein) were grown under standardized feeding and housing conditions and slaughtered in the abattoir of the Leibniz Institute for Farm Animal Biology at 18 months of age. Details of the experiment were described by Kuehn et al. (2002) and Widmann et al. (2015). Experimental procedures and animal care were carried out according to the guidelines of the German Law of Animal Protection. The protocols were approved by the Animal Protection Board of the Leibniz Institute for Farm Animal Biology as well as by the Animal Care Committee of the State Mecklenburg-Western Pomerania, Germany (State Office for Agriculture, Food Safety and Fishery; LALLF M-V/TSD/7221.3-2.1-010/03).

Carcass composition and meat quality traits were measured as described by Pfuhl et al. (2007) for bulls of the founder breeds of this F_2_-generation. Marbling traits were recorded as described by Albrecht et al. (2011b). Samples from subcutaneous fat (SCF), perirenal fat (PF), omental fat (OF), intestinal fat (IF), liver and M. longissimus dorsi (MLD) were collected immediately after slaughter, frozen in liquid nitrogen and stored at −80°C until further use. Two groups of animals (*n* = 20 each) were retrospectively assigned according to the accumulated amount of carcass fat as high (HCF; 94.2 ± 6.4 kg) and low carcass fat (LCF; 36.0 ± 6.4 kg) group for comparisons.

### Isolation of DNA and RNA, cDNA synthesis

Genomic DNA was isolated using standard procedures (phenol chloroform isopropanol precipitation with proteinase K treatment) from 30 mg liver tissues. The methods for RNA isolation from adipose tissues, muscle and liver followed standard protocols and are described in detail by Schering et al. (2017). The amount of total RNA was measured with a NanoDrop ND-1000 spectrophotometer (Peqlab, Erlangen, Germany) and the integrity of RNA was determined with an Experion Automated Electrophoresis System using the RNA StdSens analysis chip (Bio-Rad, Munich, Germany). The quality of RNA samples was considered sufficient if the RQI (RNA quality indicator) value was above 7. The iScript cDNA Synthesis Kit (Bio-Rad) was used to synthesize first strand cDNA from 100 ng total RNA of the respective tissue in 20 μl reaction volume according to manufacturer's instructions.

### Qualitative polymerase chain reaction (PCR)

Exon2C transcript of *ASIP* was detected in genomic DNA by standard PCR with specific primers (Table 1) according to Albrecht et al. (2012). The mRNA expression of *ASIP* in liver (primers in Table 2) was initially tested with a cDNA-PCR approach. Target sequences were amplified in a 10 μl reaction volume with 10 ng cDNA, 2 μM primer pairs, and PCR master mix (2×) (Fermentas, St. Leon-Rot, Germany) in a pecSTAR 96 universal thermocycler (Peqlab, Erlangen, Germany). The amplification started with an initial denaturation at 94°C for 4 min, followed by 35 cycles with 94°C for 30 s, a template specific annealing temperature for 1 min, 72°C for 1 min, and final step with 72°C for 7 min. The annealing temperatures are provided in Tables 1, 2. The PCR products were subjected to electrophoresis on 3.0% agarose gels containing Roti-GelStain (Carl Roth GmbH, Karlsruhe, Germany) and visualized under UV light (Quantum, Peqlab).

**Table 1:** Primer sequences for standard PCR.

**Primer name**	**Sequence (5′ 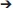 3′)**	**Genbank acc. no. position**	**Amplicon length (bp)**	**Ta (°C)**
*ASIP* Exon2C^a^		GK000013.2^b^	419	62
*for*	AAATCAACATCTCGGCTTGG	3–22		
*rev*	CTTTTCTGGGTGCCTGATGT	421–402		
*ASIP* genomic^a^		GK000013.2^b^	430	62
*for*	AAATCAACATCTCGGCTTGG	3–22		
*rev*	AAAAGGAAAGTGCGGAGGAG	7,089–7,070		
*MC4R*_SNP1		AC_000181.1^c^	755	57.5
*for*	AAAATCCTGAATGTTGCCTGG	3,056–3,036		
*rev*	TCCCAATTCAGAGGCAGAAG	2,282–2,301		
*MC4R*_SNP2		AC_000181.1^c^	601	59
*for*	TTCTGCCTCTGAATTGGGATTAC	2,300–2,278		
*rev*	GGCTGGGTAGAGTTCATTTTGG	1,700–1,721		
*MC4R*_SNP3		AC_000181.1^c^	541	58
*for*	TCGTTTGGGGCAAGTCAAG	1,804–1,786		
*rev*	GATAGTGAAGTACCTGTCCACC	1,264–1,285		
*MC4R*_SNP4		AC_000181.1^c^	736	59
*for*	CTCGGTGATCTGTAGCTCCTT	1,339–1,318		
*rev*	CACTCCATGCCCTACACAGA	604–623		
*MC4R*_SNP5		AC_000181.1^c^	544	57.5
*for*	TGAAACAGTGCCCAGTCTT	558–540		
*rev*	TAATCCCAAATTGCCTGTGAG	15–35		
*ATRN*_SNP		AC_000170.1^d^	600	57
*for*	CAGCGAAACTTGGGGTAGAGT	3,416–3,396		
*rev*	GGCCTTCCAGCTCTAGTCG	2,817–2,835		

a *Adopted from Albrecht et al. (2012)*.

b *Nucleotide positions refer to partial sequence of GK000013.2 starting at nt 64,212,950 (i.e., nt 1 in this table = nt 64,212,950)*.

c *Nucleotide positions refer to partial sequence of AC_000181.1 starting at nt 59,670,000 (i.e., nt 1 in this table = nt 59,670,000)*.

d *Nucleotide positions refer to partial sequence of AC_000170.1 starting at nt 52,190,000 (i.e., nt 1 in this table = nt 52,190,000)*.

**Table 2:** Primer sequences used for RT-qPCR.

**Primer name**	**Sequence (5′ 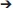 3′)**	**Genbank acc. no. position**	**Amplicon length (bp)**	**Ta (°C)**
*ASIP*^a^		NM_206843.2	181	60
*for*	TACCTTGCTGGTCTGCCTGT	200–219		
*rev*	CTTTTCCGCTTCATTTCTGC	380–361		
*MC1R*^a^		NM_174108		
*for*	CTGCACTCCCCCATGTACTA	488–507	233	60
*rev*	ATGGAGATGTAGCGGTCCAC	720–701		
*MC4R*^a^		NM_174110.1	163	60
*for*	CTGATCGGGGTCTTTGTTGT	961–980		
*rev*	GGGCATAAATCAGAGGGTCA	1,123–1,104		
*ATRN*		NM_173995.3	245	60
*for*	CAGTAATTTCACCTGGCCCATC	3,780–3,801		
*rev*	CATCTGTTTCCAAGGCGACAT	4,024–4,004		
*B2M*		NM_173893	184	60
*for*	CAGCTGCTGCAAGGATGG	181–204		
*rev*	ATTTCAATCTGGGGTGGATG	417–394		
*TOP2B*		XM_010820383.1	173	60
*for*	AAGAAAACAGCACCGAAAGG	4,519–4,538		
*rev*	AGGTCTGAGGGGAAGAGGT	4,691–4,673		
*UXT*		NM_001037471	70	60
*for*	TCATGGCGACGCCCCCTAAAC	30–50		
*rev*	AAAGCCTCGTAGCGCAGCACT	99–79		
*RPS9*		NM_001101152.2	152	60
*for*	GCTGATCGGCGAGTATGGGCT	146–166		
*rev*	GCCGCCGCAACAGGGCATTA	297–278		
*FABP3*		NM_174313.2	154	60
*for*	GCGTTCTCTGTCGTCTTTCC	7–26		
*rev*	CTTGGTCATATTGCCCACCT	160–141		
*FABP4*		NM_174314.2	174	60
*for*	GGATGGAAAATCAACCACCA	354–373		
*rev*	TGGACAACGTATCCAGCAGA	527–508		

a *Adopted from Albrecht et al. (2012)*.

### Quantitative PCR (qPCR)

Expression levels of investigated genes were analyzed by RT-qPCR (iCycler MyiQ 2 with iQ detection system, Bio-Rad) in duplicates as described by Schering et al. (2017). Briefly, 10 ng cDNA template, 2 μM of the respective forward and reverse primers, and 5 μl SYBR Green Supermix (Bio-Rad) were used in 10 μl reaction volumes. Primers (Table 2) were designed with Primer 3web (Version 4.0.0, http://primer3.ut.ee/) and synthesized by Sigma-Aldrich. Products were amplified after initial denaturation (95°C for 3 min) in 45 cycles (95°C for 10 s, 60°C for 30 s, 70°C for 45 s). The expression values for all tissues were normalized to each two reference genes: beta-2-microglobulin (B2M) and ubiquitously-expressed transcript (UXT) for adipose tissues; UXT and ribosomal protein S9 (RPS9) for liver tissue; topoisomerase II beta (TOP2B) and B2M for muscle tissue. Crossing point (C_P_) values were adapted manually in the iQ5 Software (Version 2.1.97.1001, Bio-Rad) to receive comparable values for the standard curves on each plate. Standard curves were generated to account for the efficiency of amplifications. They were calculated from serial dilutions (for *ASIP*: 1:1, 1:2, 1:5, 1:10, 1:20, for other genes: 1:1, 1:10, 1:50, 1:100, 1:500). Normalizations were calculated using the efficiency-corrected ΔΔC_p_ method (Pfaffl et al., 2002). The significance of differences in two group comparisons was tested with the REST algorithm (Version 2.0.13, QIAGEN, Hilden, Germany). Normalized relative quantities (NRQ, Hellemans et al., 2007) were used if more than two groups were compared and for calculation of Pearson correlation coefficients to determine relationships between mRNA abundance and phenotypic traits.

### Genotypes at the bovine MC4R locus and ATRN locus

Standard PCR for *MC4R* and *ATRN* was performed with genomic DNA and specific primers (Table 1) in samples of HCF bulls (*n* = 20) and LCF bulls (*n* = 20). For primer pairs of *MC4R*, the PCR reaction system was as described above. In the PCR reaction system of *ATRN*, 5% DMSO was added to improve amplification because of the high GC content of the target sequence of *ATRN*. The amplicons were purified with the High Pure PCR Product Purification Kit (Roche Diagnostics, Mannheim, Germany). The PCR products were analyzed on an ABI PRISM 310 Genetic Analyzer (Applied Biosystems, Darmstadt, Germany). Sequences were analyzed with CLC Main Workbench (v. 7.7, CLCbio, Aarhus, Denmark).

### Morphology of adipocytes within MLD and SCF

Sections of MLD and SCF were cut (12 and 20 μm thick, respectively) with a cryostat microtome (CM3050S, Leica, Germany) and stained with hematoxylin/eosin (H/E) using standard protocols. Adipocytes size was measured using the interactive measurement module of the Cell^∧^D image analysis software (OSIS, Münster, Germany) as described by Albrecht et al. (2011b). The image analysis system was equipped with a Jenaval microscope (Carl Zeiss, Jena, Germany) and an Altra20 CCD camera (OSIS, Münster, Germany). At least 200–300 adipocytes per sample were randomly selected and measured.

### Collection of muscle fibers and adipocytes by laser capture microdissection (LCM)

The method for sampling muscle fibers and adipocytes from unstained, dehydrated cross-sections of MLD by LCM was described by Albrecht et al. (2011a). Briefly, MLD samples were cut into 12 μm thick serial sections with a cryostat microtome (CM3050S, Leica, Germany). Sections were transferred to a membrane slide (Palm, Bernried, Germany) and were shortly air-dried (10 s). Slides were placed successively in 96% and 2 × in 100% ethanol at room temperature for 30 s each and then in xylene for 5 min to dehydrate the tissue. Slides were finally air-dried under a fume hood for 5 min and were immediately used for sampling of muscle fibers or adipocytes with a Palm MicroBeam LCM device (Palm). About 600 muscle fibers and 300 adipocytes per sample were collected in adhesive caps (Palm) and lysed in buffer RLT (RNeasy Micro Kit, Qiagen). The lysates were stored at −80°C until further extraction of total RNA with the RNeasy Micro Kit (Qiagen). The total volume of RNA (14 μl) was used for cDNA synthesis using iScript cDNA Synthesis Kit (Bio-Rad).

### Antibodies against ASIP, MC1R, MC4R, AND ATRN, and recombinant protein

Antibodies against ASIP were produced by Novus Biologicals (NBP2-14323, Bio-Techne, Wiesbaden, Germany) and Cusabio (CSB-PA002212LA01HU, Biotrend, Cologne, Germany). Both antibodies are polyclonal rabbit anti-human ASIP, but were expected to cross-react with the bovine protein due to high sequence homology in the antigen region. The polyclonal antibodies against MC1R (ELA-ENT2673, Elabscience) and ATRN (BYT-ORB155769, Biorbyt) were also generated in rabbit and purchased from Biozol (Eching, Germany). The polyclonal rabbit anti-human MC4R antibody (ABIN2704972, Cohesion Bioscience) and the respective blocking peptide were purchased from antibodies-online (Aachen, Germany). Recombinant bovine ASIP from Cusabio (CSB-EP639982BO, Biotrend) was produced in E. coli with a his-sumo-tag and was used as positive control and for blocking of specific binding of the ASIP antibodies. Before using as a positive control in western blots, the tag was removed by incubation of the protein with sumo-protease (Invitrogen) according to manufacturer's instructions.

### Western blotting

Total protein was extracted from SCF, liver, and muscle tissue with CelLytic MT lyses reagent (Sigma-Aldrich, Munich, Germany) and protease inhibitor following manufacturer's instructions. Protein was mixed with loading buffer and denatured at 95°C for 5 min before loading on Criterion TGX 12% gel (Bio-Rad). Two molecular weight markers (PageRuler, Thermo Scientific; Triple Color Protein Standard III, SERVA) were used to determine the molecular weight of the protein bands. After electrophoresis, proteins were transferred to a polyvinylidene difluoride (PVDF) membrane (Tans-Blot Turbo transfer pack, Bio-Rad) with a semi dry blotter (Trans-Blot, Bio-Rad). Coomassie staining (Brilliant Blue R-250, Carl Roth) was performed to verify proper transfer of the protein to membranes. Membranes were blocked for 1 h with 5% non-fat dry milk or 10% Roti-Block (Carl Roth) in Tris-buffered saline (TBS). Then the membranes were incubated with primary antibodies (ASIP 1:5,000, MC1R and MC4R 1:2,000, ATRN 1:1,000) over night at 4°C. To test the specificity of ASIP antibody, recombinant protein was also prepared as a loading sample. Two parallel blots were incubated with the antibody or with the antibody blocked with the recombinant protein before incubation. Membranes were incubated with HRP conjugated Trueblot rabbit IgG (1:25,000; Biomol, Hamburg, Germany) secondary antibody. The antibody label was detected with highly sensitive chemiluminescence substrate (Super Signal West Femto Substrat, Thermo Scientific). Chemiluminescence was recorded with a Chemocam HR-16 imager (INTAS, Göttingen, Germany) and quantified with Labimage 1D software (Kapelan Bio-Imaging, Leipzig, Germany). Band intensities were normalized to total protein abundance in the respective lane measured in the Coomassie stained blot image (Figure S1).

### Immunohistochemical analysis

Different bovine tissues were cryo-sectioned (thickness liver: 10 μm; MLD: 12 μm; SCF 25 μm) using a Leica CM3050 S (Leica, Bensheim, Germany) cryostat microtome. Sections were air dried, fixed with 4% paraformaldehyde, and washed three times with PBS. Unspecific binding of the secondary antibody was blocked using 10 % goat serum in PBS for 15 min. Sections were incubated for 2 h at room temperature in a humidity chamber with the same primary antibodies as used for western blots, diluted 1:100 in PBS. Specific binding of primary antibody was detected with the respective goat anti rabbit IgG secondary antibody labeled with Alexa Fluor 488 (Molecular Probes, Eugene, USA). Nuclei were counterstained with 1 μg/ml Hoechst 33258 (Sigma-Aldrich, Munich, Germany). Slides were covered using ProLong Diamond Antifade Mountant (Thermo Scientific) and appropriate cover-slips. Negative controls were incubated either omitting the primary antibody or blocking the primary antibody with the respective peptide or recombinant protein. No unspecific binding of the secondary antibody, but some unspecific binding of the primary antibodies was detected. Immunofluorescence was visualized with a Nikon Microphot SA fluorescence microscope (Nikon, Duesseldorf, Germany) and an image analysis system equipped with CELL^∧^F software and a CC-12 high resolution color camera (OSIS, Muenster, Germany).

### Statistical analyses

Data were analyzed with SAS statistical software (Version 9.2, SAS Inst., Cary, USA). Pearson correlation coefficients using the CORR procedure in SAS were calculated to obtain relationships between gene expression and carcass traits. Phenotypic traits and NRQ values were analyzed with ANOVA with fixed factor group and Fishers test of Least Significant Differences. The mixed model was used to test the influence of sire line within each group. Neither significant influence of this effect nor of the group × sire line interaction effect could be detected. The NRQ values of ASIP in adipose tissues were analyzed with ANOVA using the MIXED procedure with fixed factor group and repeated factor tissue (with unstructured covariance structure) and group × tissue interaction. The Tukey-Kramer-correction was used to control the Family-wise Error Rate (referred to as P_adj_). Differences were considered as significant if *P*_adj_ ≤ 0.05.

## Results

### Relationships between carcass traits and *ASIP* expression

Bulls of the F_2_-generation of a Charolais × Holstein cross were used in this study. They were comprehensively phenotyped and varied widely in body weight and composition. Despite of a similar genetic background and identical feeding and housing conditions, bulls accumulated variable amounts of fat in the carcass, in different fat depots, and within the muscle (Table 3). Thus, the material appears to be useful to investigate associations between the expression of *ASIP* and its receptors with body composition.

**Table 3:** Selected phenotypic traits of 246 F_2_-generation bulls (Charolais × Holstein cross) slaughtered at 18 months of age.

**Trait**	**LSmean**	**Min**	**Max**	***SD***
Age, days	547	538	559	3
Body weight, kg	691	470	818	58
Average daily gain, kg	0.68	0.44	0.82	0.06
Cold carcass weight, kg	392.1	264.9	472.8	34.9
Liver, kg	7.29	4.49	9.70	0.91
Meat, %	77.10	72.01	81.29	1.57
Bones, %	14.74	11.03	19.05	1.61
Tendons, %	2.47	1.68	3.30	0.34
Carcass fat, kg	64.45	24.27	107.26	16.01
Carcass fat, %	16.37	6.98	27.99	4.04
Carcass protein, %	14.52	11.94	16.40	1.23
Subcutaneous fat of right half, kg	11.19	3.60	21.80	3.08
Subcutaneous fat, %	5.68	2.19	11.74	1.48
Intestinal fat, kg	9.47	1.01	26.80	3.42
Intestinal fat, %	1.37	0.15	3.44	0.47
Omental fat, kg	16.83	5.50	35.60	4.62
Omental fat, %	2.44	0.95	4.85	0.64
Perirenal fat, kg	16.34	5.26	29.62	4.31
Perirenal fat, %	2.36	0.91	4.07	0.60
M. longissimus weight, kg	8.07	5.14	10.23	0.92
IMF^a^ of M. longissimus, %	3.37	0.63	10.68	1.71
Marbling fleck area percentage, %	5.62	0.90	15.96	2.71
Number of marbling flecks	581	109	1160	200
Distance between marbling flecks, mm	1.90	1.33	3.85	0.34

a *IMF, intramuscular fat content*.

**Figure 1:**
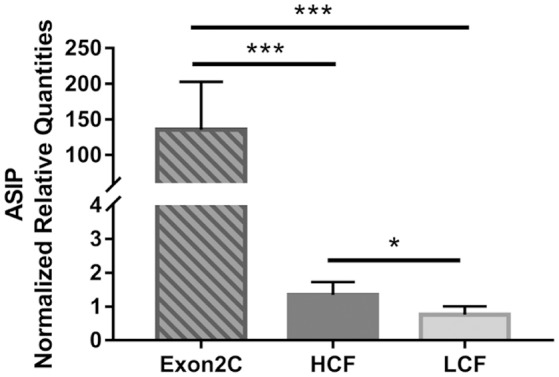
Normalized mRNA abundance of ASIP in subcutaneous fat of bulls from three groups (Exon2C, *n* = 17; HCF, *n* = 20; LCF, *n* = 20). Data are expressed as mean + SEM. ^*^,^***^ Indicate significant differences with *P* < 0.05 and *P* < 0.001, respectively. HCF, high carcass fat; LCF, low carcass fat.

The mRNA abundance of *ASIP* was determined in SCF of all 246 bulls for correlations with carcass traits. A markedly increased amount of *ASIP* mRNA was observed in 17 of these bulls (Figure 1). Standard PCR test for existence of a transposon-derived transcript (Exon2C) as cause for *ASIP* overexpression confirmed that these 17 bulls were heterozygous for Exon2C (Figure S2). It could be verified that this transposon was introduced by one Charolais sire into our experimental population.

Further analyses were conducted in three retrospectively assigned groups of bulls. Two groups with highest or lowest amount of carcass fat were investigated as high carcass fat group (HCF) and low carcass fat group (LCF), respectively, to compare gene expression in different adipose tissues, liver, and muscle of bulls with maximum difference in body fat deposition. Since there is no report about effects of *ASIP* Exon2C on fat deposition in cattle, these bulls were separately analyzed as Exon2C group. None of the 17 bulls overexpressing *ASIP* (Exon2C group) belongs to the HCF or LCF group.

Correlation analysis between NRQ values of *ASIP* and carcass traits was conducted with 229 bulls without Exon2C to avoid bias caused by extremely high expression of *ASIP* in this group. Normalized mRNA values of SCF showed low but significant correlations with body weight, daily gain, liver weight, and fat deposition related traits (Table 4). However within the Exon2C group, associations between these traits were not detected.

**Table 4:** Significant Pearson correlation coefficients between *ASIP* mRNA abundances (NRQ, normalized relative quantities) in subcutaneous fat and traits of F_2_-generation bulls at 18 months of age.

**Trait**	***ASIP*** **normalized mRNA values (*****n*** = **229)**	
	***r***	***P*-value**
Body weight, kg	0.209	0.0015
Average daily gain, kg	0.231	0.0004
Liver, kg	0.210	0.0014
Carcass fat, kg	0.293	<0.0001
Subcutaneous fat, kg	0.285	<0.0001
Intestinal fat, kg	0.174	0.0084
Omental fat, kg	0.193	0.0033
IMF^a^ of M. longissimus, %	0.228	0.0005
Marbling fleck area percentage, %	0.151	0.0268
Number of marbling flecks	0.227	0.0008
Distance between marbling flecks, mm	−0.170	0.0134

a *IMF, intramuscular fat content*.

**Figure 2:**
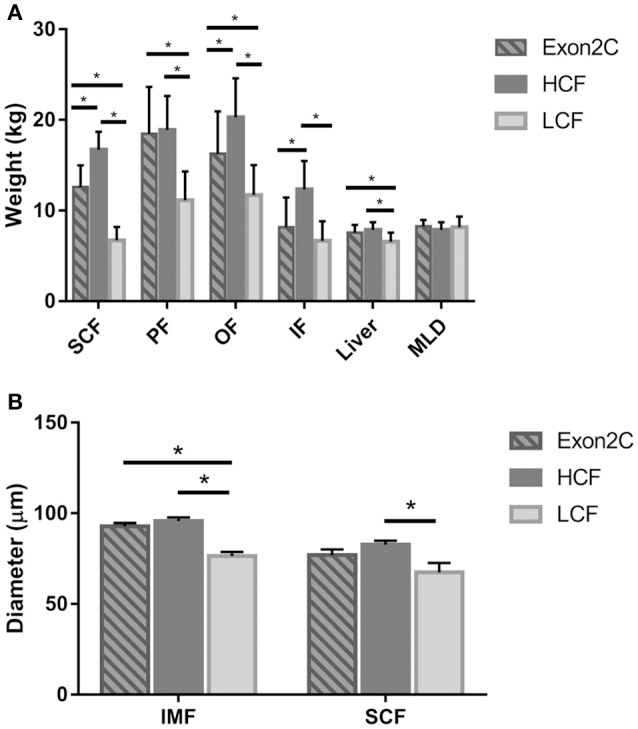
Weights of investigated tissues **(A)** and size of intramuscular and subcutaneous adipocytes **(B)** of bulls from three groups (Exon2C, *n* = 17; HCF, *n* = 20; LCF, *n* = 20). Data are expressed as mean + SEM. ^*^ Indicates significant differences with *P* < 0.05. HCF, high carcass fat; LCF, low carcass fat; SCF, subcutaneous fat; PF, perirenal fat; OF, omental fat; IF, intestinal fat; MLD, M. longissimus dorsi; IMF, intramuscular fat.

**Figure 3:**
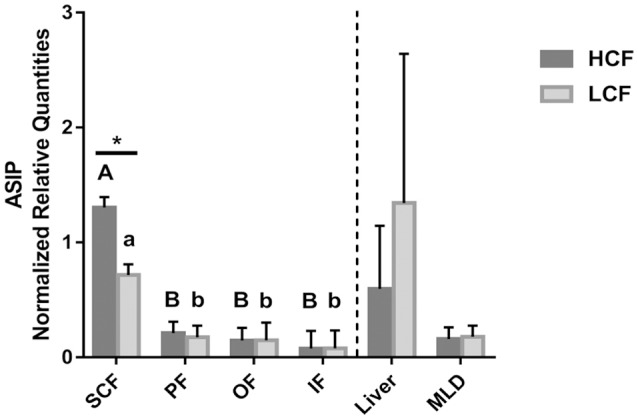
Abundance of *ASIP* mRNA in different adipose tissues, liver and muscle of HCF and LCF bulls (*n* = 20 each). Data are expressed as mean + SEM. ^*^ Indicates a significant difference between groups in SCF with *P* < 0.05; A, B indicate significant differences between adipose tissues within HCF bulls (*P* < 0.01). a, b indicate significant differences between adipose tissues within LCF bulls (*P* < 0.01). HCF, high carcass fat; LCF, low carcass fat; SCF, subcutaneous fat; PF, perirenal fat; OF, omental fat; IF, intestinal fat; MLD, M. longissimus dorsi.

Next, the phenotypic traits and mRNA abundance of *ASIP* in different adipose tissues, liver, and muscle were determined for the three assigned groups of bulls (Figures 2, 3 and Table S1). All adipose tissues and the liver were heavier in bulls of the HCF group than in the LCF group (Figure 2A). Furthermore, bulls from the Exon2C group had more SCF, PF, OF, and heavier livers than the LCF group, whereas the weights of SCF, OF, and IF were lower than in the HCF group (Figure 2A). The size of intramuscular and subcutaneous adipocytes differed significantly between groups (Figure 2B). Bulls of the LCF group had smallest intramuscular adipocytes among groups and smaller (*P* < 0.001) subcutaneous adipocytes than HCF bulls (*P* = 0.016).

The mRNA of *ASIP* was detected in muscle, liver, and adipose tissues of bulls of all three groups. Increased amounts of *ASIP* mRNA were observed in all investigated tissues in the Exon2C group (data not shown). All Exon2C bulls but only some of HCF and LCF bulls had measurable amounts of *ASIP* mRNA in liver (Figure S3), OF, and IF (liver: *n*_HCF_ = 8, *n*_LCF_ = 10; OF: *n*_HCF_ = 14, *n*_LCF_ = 7; IF: *n*_HCF_ = 7, *n*_LCF_ = 7). This indicates ectopic expression of *ASIP* if Exon2C is included.

Apart from the Exon2C group, a significant difference between HCF and LCF bulls for *ASIP* mRNA abundance was solely detected in SCF, with about 1.6-fold higher (*P* < 0.05) value in HCF bulls (Figure 3). Higher mRNA levels were measured in SCF compared to other adipose tissues in both groups of bulls (Figure 3). There was no difference in *ASIP* mRNA abundance between HCF and LCF bulls in liver and MLD tissue.

### Expression of *ASIP* in isolated adipocytes and muscle fibers

To clarify whether the mRNA of *ASIP* is expressed in specific cell types of bovine muscle tissue, we collected muscle fibers and intramuscular fat cells separately from muscle tissue by laser capture microdissection (LCM). The expression of *FABP3* and *FABP4* was used as marker for the purity of the respective cell collections. As expected, *FABP3* mRNA was exclusively expressed in muscle fibers and the mRNA of *FABP4* could only be detected in adipocytes. The mRNA of *ASIP* was detected in adipocytes and muscle fibers if Exon2C was present (Table 5). However, *ASIP* mRNA was not detected in separated cells of a HCF or LCF bull (Table 5), despite expression in whole muscle tissue.

**Table 5:** Detection of mRNA of *ASIP* and cell type markers in laser microdissected adipocytes (AC) and muscle fibers (MF).

**Gene**	**Exon2C**		**HCF**		**LCF**	
	**AC**	**MF**	**AC**	**MF**	**AC**	**MF**
*FABP3*	ND	21.31	ND	25.57	ND	24.91
*FABP4*	19.89	ND	21.06	ND	22.78	ND
*ASIP*	29.45	25.52	ND	ND	ND	ND

*Cells were isolated from longissimus muscle tissue sections of one bull of each group (Exon2C, HCF, high carcass fat; LCF, low carcass fat). Original C_p_-values are presented as obtained by RT-PCR (ND, not detected)*.

### Receptor expression

Expression of receptors for ASIP in different adipose tissues, muscle, and liver is a prerequisite for auto-, para-, and endocrine action of ASIP. Therefore, the mRNA of *MC1R, MC4R*, and *ATRN* was determined in these tissues. The *MC1R* mRNA could be detected in SCF, liver, and MLD, but was not differentially expressed among Exon2C, HCF, and LCF bulls (data not shown). In other investigated adipose tissues, *MC1R* mRNA was detected only in a few samples so that it was not possible to analyze those data statistically.

**Figure 4:**
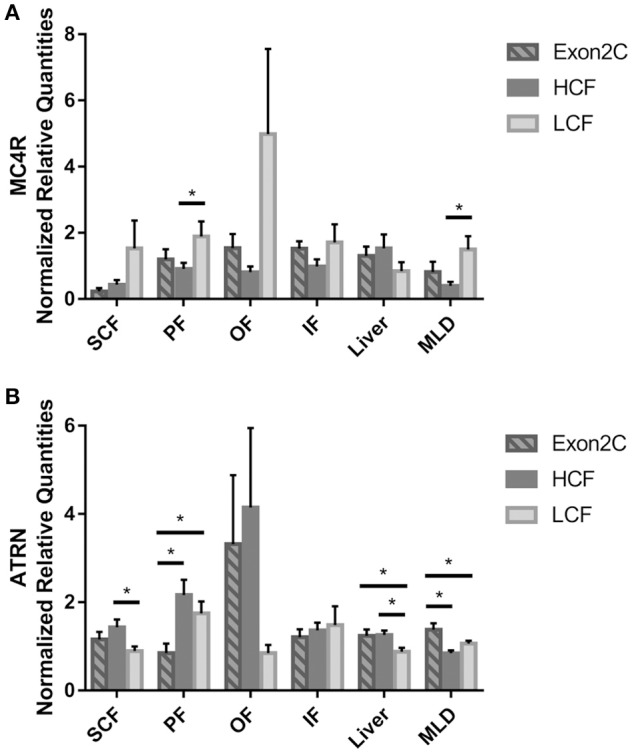
Abundances of *MC4R* **(A)** and *ATRN* **(B)** mRNA in different tissues of three groups of bulls (Exon2C, *n* = 17; HCF, *n* = 20; LCF, *n* = 20). Data are expressed as mean + SEM. ^*^ Indicates significant differences with *P* < 0.05. HCF, high carcass fat; LCF, low carcass fat; SCF, subcutaneous fat; PF, perirenal fat; OF, omental fat; IF, intestinal fat; MLD, M. longissimus dorsi.

Differences in expression of the other two receptor genes could be observed between HCF and LCF bulls in several tissues. The expression level of *MC4R* was lower in PF and MLD of HCF compared to LCF bulls (Figure 4A). There was no difference between Exon2C and HCF or LCF bulls (*P* > 0.05). In SCF and MLD, the mRNA of *MC4R* was only detectable in a part of the bulls of three groups (SCF: *n*_HCF_ = 9, *n*_LCF_ = 9, *n*_Exon2C_ = 5; MLD: *n*_HCF_ = 11, *n*_LCF_ = 13, *n*_Exon2C_ = 10). The amount of *ATRN* mRNA in SCF and liver was higher (*P* < 0.05) in HCF compared to LCF bulls (Figure 4B). Bulls of the Exon2C group had lower *ATRN* mRNA values than HCF and LCF bulls in PF, whereas it was higher (*P* < 0.05) in muscle (Figure 4B). Moreover, LCF bulls had less (*P* < 0.05) *ATRN* mRNA than Exon2C and HCF bulls in liver (Figure 4B).

Intramuscular adipocyte size and *MC4R* mRNA abundance in MLD were negatively correlated (*r* = −0.46, *P* < 0.001). However, correlations between adipocyte size in SCF and mRNA expression of *ASIP* or one of the receptors were not observed (*P* > 0.1).

### SNPs as possible reason for expression differences

Bovine *MC4R* locus and *ATRN* locus were screened for SNPs, especially in regions with potential effects on gene expression. Screening of the bovine MC4R locus comprised 1100 base pairs (bp) 5′ of the transcription start, the complete coding region, and 700 bp 3′ of the stop codon. This gene has no intron. For the *ATRN* gene, 500 base pairs (bp) 5′ of the transcription start were sequenced. No SNP was detected in either regulatory region thus excluding effects of sequence variation within or close to the genes on expression differences.

The alignment however, revealed two SNPs (c. 1069 C>G and c. 1202 G>A) in the coding region of *MC4R* and one SNP (g.-236 G>C) upstream of the 5′ UTR of *ATRN* locus. The SNP c. 1069 C>G of *MC4R* has been described before, whereas the other SNPs, *MC4R* c. 1202 G>A and *ATRN* g.-236 G>C, are novel. Allele frequencies were not different between HCF and LCF groups in all SNPs.

### Protein abundance of ASIP and its receptors

**Figure 5:**
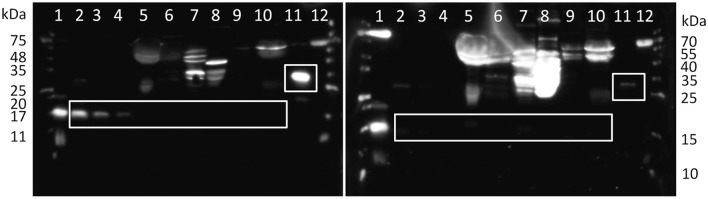
Western blot detection of bovine ASIP. White boxes indicate specific, blockable bands of recombinant ASIP and the respective size in tissues. The right panel was incubated with antibody previously blocked with the antigen peptide to determine unspecific bindings. Lanes 1 and 12: molecular weight markers, lanes 2–4: 50, 25, 12.5 ng recombinant bovine ASIP after tag removal in PBS, respectively, lanes 5–8: 50 μg of total protein from bovine albumin depleted plasma, subcutaneous fat (SCF), liver, M. longissimus dorsi, respectively, lane 9–10: 50 μg of total protein from bovine SCF and albumin depleted plasma, respectively, after deglycosylation, lane 11: 50 ng recombinant bovine ASIP with SUMO-tag.

Bovine ASIP contains 133 amino acids (UniProtKB entry: Q29414), and has 77% similarity with mouse ASIP and 75% with human ASIP. There are currently no experimentally derived data available on this protein in cattle. Commercial antibodies against human ASIP were tested for cross reactivity with bovine protein. The recombinant protein could be recognized by the antibody of Cusabio, but not by the antibody of Novus Biologicals. Specific bands were observed for recombinant ASIP with sumo tag at ~30 kDa and after removal of the tag at ~15 kDa (Figure 5, lanes 2–4), in accordance with the theoretical size. The band intensity corresponded to the protein amount in the respective lanes (Figure 5). However, a specific band for ASIP could not be detected in bovine plasma and tissue samples. There were only several unspecific bands, as confirmed by blocking of the antibody binding with recombinant protein before incubation. All bands in tissues and plasma were enhanced if specific binding of the primary antibody was inhibited, whereas the specific bands of recombinant protein disappeared (Figure 5). Protein abundance in bovine tissues is probably below the detection limit (< 12.5 ng), since there was no specific ASIP band observed; even in tissue of Exon2C bulls (data not shown). This indicates that overexpression of this specific ASIP transcript has no consequence for the protein abundance.

**Figure 6:**
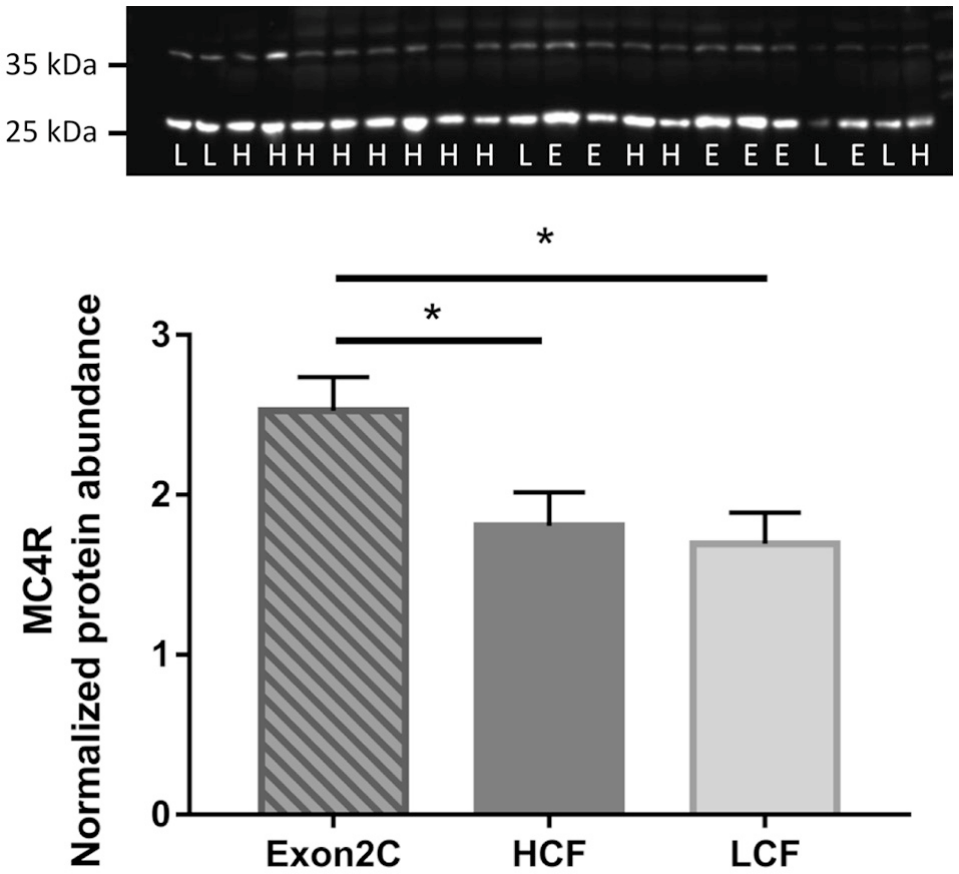
Quantification of MC4R protein abundance in liver of three groups of F_2_-generation bulls at 18 months of age (Exon2C, *n* = 17; HCF, *n* = 20; LCF, *n* = 20). A representative western blot image is shown above. H = HCF, high carcass fat; L = LCF, low carcass fat; E, Exon2C. ^*^ Indicates significant higher value in Exon2C bulls with *P* < 0.05.

Western blot analysis of MC4R revealed two bands in liver, one at ~25 kDa and the other at ~35 kDa, which corresponds to the theoretical size of MC4R. Both bands disappeared if the antibody was preincubated with the antigen peptide for blocking of specific bindings. However, only unspecific bands were observed in muscle and SCF of bulls (Figure S4), in contrast to mouse tissue. Quantification of MC4R protein abundance in liver revealed highest values for Exon2C bulls (*P* < 0.05, Figure 6). No differences between groups were observed, if only the band of ~35 kDa was analyzed.

The antibodies against MC1R and ATRN did not recognize specific bands at the theoretical size of the proteins in bovine samples. Therefore, quantification was not possible.

### Protein localization in bovine tissues

**Figure 7:**
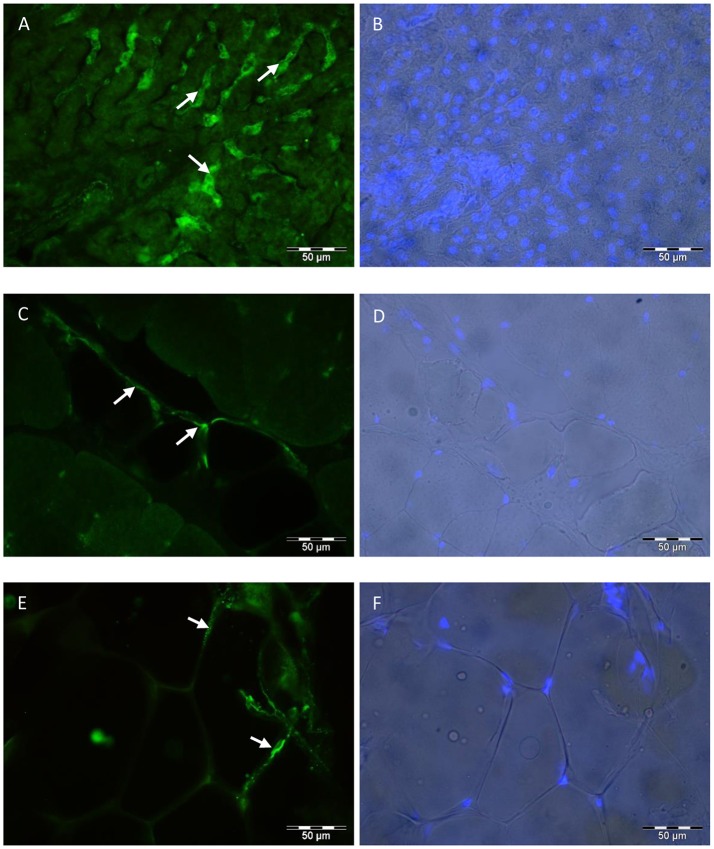
Localization of ASIP in bovine tissues. The polyclonal rabbit anti-ASIP antibody was detected with an Alexa Fluor 488 labeled goat anti rabbit IgG secondary antibody in bovine liver **(A)**, MLD **(C)**, and SCF **(E)**. Nuclei were stained with Hoechst 33248 and overlaid to the brightfield image of liver **(B)**, MLD **(D)**, and SCF **(F)**, respectively. Arrows indicate specific staining in stellate cells **(A)** or in and around adipocytes **(C,E)**. SCF, subcutaneous fat; MLD, M. longissimus dorsi.

In muscle and SCF, ASIP was localized in or around adipocytes with immunohistochemistry (Figure 7). Muscle fibers were not stained for ASIP, not even in muscle tissue of Exon2C bulls (data not shown). Positive cells in the liver were probably stellate cells (Figure 7). The staining pattern in liver corresponded to the staining with the MC4R and ATRN antibodies (data not shown). The MC4R and ATRN were not detectable in muscle and SCF (data not shown). No specific signal could be observed in none of the tissues when using the antibody against MC1R (data not shown).

## Discussion

The present study investigated whether ASIP and its receptors may be involved in variation of fat deposition within F_2_-generation bulls of a Charolais × Holstein cross kept under identical feeding and housing conditions. A group of 17 bulls was detected with ectopic expression of *ASIP* paralleled by the abundance of a full-length long interspersed nuclear element (LINE-1; L1-BT element; Exon2C) inserted in the *ASIP* gene. Such L1 elements are able to alter expression of affected genes in amount and splicing with possible consequences for the protein translation (Kines and Belancio, 2012). The L1 element in bovine *ASIP* (Exon2C) was first found in Normande and described by Girardot et al. (2006). We found in our former study with Japanese Black (JB), Holstein, and Charolais steers that this Exon2C was abundant in JB and sporadic in Charolais but not in Holstein (Albrecht et al., 2012). It could be confirmed in the current study that the 17 bulls overexpressing *ASIP* were heterozygous for Exon2C and all bulls belonged to the same sire line. This transposon caused *ASIP* mRNA overexpression in all investigated tissues of our F_2_-generation bulls. Furthermore, the expression of *ASIP* mRNA was detectable in both muscle and fat cells if Exon2C was present. However, consequences of *ASIP* overexpression on protein abundance or localization could not be detected by western blot or immunohistochemistry, respectively. The protein abundance was below the detection limit of the western blot even in these bulls. Further studies are necessary to clarify whether overexpression leads to increased translation into protein.

In lethal yellow (A^y^) mouse, a mutation in a non-coding exon of agouti gene caused overexpression of *Asip* mRNA and protein (Bultman et al., 1992). Beside yellow hair, those A^y^ mice have an obesity phenotype, in contrast to wildtype animals. Human *ASIP* polymorphisms in a non-coding region, which can cause *ASIP* overexpression like in mice and bovine, have not been reported yet. Overexpression of *ASIP*, as observed in bulls of the Exon2C group of our study, did not cause ectopic fat accretion. Bulls of the Exon2C group stored more fat in SCF, PF, and OF than LCF bulls, but not more than bulls of the HCF group. The inconsistency between *ASIP* mRNA and protein level can give rise to this phenomenon. Moreover, compared to A^y^ mice, there could be an alternative regulation mechanism on *ASIP* expression in cattle with Exon2C transcript. Sadkowski et al. (2014) reported 32.6-fold and 2.2-fold higher *ASIP* expression levels in Hereford bulls and Holstein-Friesian bulls (marbled meat) compared to Limousin bulls (lean meat), respectively. It should be considered that this transposon could also be involved in extremely high expression of *ASIP* in Hereford bulls.

Agouti is normally expressed in hair follicles and testis during different life periods in wild mouse (Bultman et al., 1992). In humans, *ASIP* is widely expressed in tissues, including adipose tissue, skin, heart, testis, ovary, and at lower levels in liver and kidney (Wilson et al., 1995). Cattle show the similar expression pattern of *ASIP* like human. Several studies detected *ASIP* mRNA in adipose tissue, M. longissimus, skin, heart, testes, ovary, and kidney in bovine (Sumida et al., 2004; Girardot et al., 2006; Graphodatskaya et al., 2006; Albrecht et al., 2012). The current study tested the expression level of *ASIP* mRNA in SCF, PF, OF, IF, MLD, and liver of F_2_-generation bulls and found that *ASIP* was stably expressed in SCF, PF, MLD, whereas it was not ubiquitously expressed in all OF, IF, and liver samples. Furthermore, among investigated adipose tissues, the mRNA level of *ASIP* was significantly higher in SCF. This may indicate that compared to other adipose tissues, SCF could contribute significantly to secreted and probably circulating amount of ASIP in bovine.

Irrespective of Exon2C bulls, there was a low, positive correlation between *ASIP* mRNA abundance of SCF and fat related traits in our F_2_-generation bulls. The expression level of *ASIP* was significantly higher in HCF bulls compared to LCF bulls. Similar associations were described between *ASIP* expression and body composition in human. Norman et al. (1998) reported *ASIP* as one of the candidate genes which show the linkage to obesity and energy metabolism in Pima Indians. Voisey et al. (2002) found that there was a negative correlation in men, whereas a positive relationship in women between *ASIP* mRNA level and body mass index. Similarly with our western blot result in bovine plasma, ASIP could not be detected in serum derived from obese and lean men and women (Voisey et al., 2002). Voisey et al. (2002) suggested that there is no ASIP circulating in human and ASIP could only act within adipose tissue. However, own RNA-sequencing data in cattle of the same experiment (not shown) and data from human adipose tissue (Xue and Zemel, 2000) suggest that ASIP is a low abundance protein also in bovine. If similar amounts of the protein are present in bovine SCF as measured and described by Xue and Zemel (2000), about 1 ng ASIP can be expected per lane in our western blot. This is below the detection limit, as indicated by the dilution series of recombinant protein (Figure 5). However, it could still circulate in hormone like concentrations and act systemically in bovine and human. Xue and Zemel (2000) demonstrated a positive relationship between ASIP protein abundance and fatty acid synthase activity in human adipose tissue and concluded that ASIP can modulate the lipid metabolism of adipocytes. Protein localization in lipid storing stellate cells in liver, with low or without measurable mRNA expression, and in adipocytes suggests, in concordance with the correlation analysis between *ASIP* mRNA level and fat related traits, that ASIP may have effects on adipocytes and fat deposition also in cattle.

Similar to our former study (Albrecht et al., 2012), ASIP was localized in adipocytes and liver cells, but in contrast to that study, it was not localized in nuclei of different cell types. Different antibodies were used in these studies and both were able to detect recombinant bovine ASIP, as shown in western blots, but differed obviously in their cross reactivity leading to different staining patterns. Therefore, only those results which agree between both antibodies were seen as reliable, like localization in and around adipocytes, and in stellate cells in the liver.

As prerequisite for peripheral effects of ASIP, the expression of potential ASIP receptors, MC1R, MC4R, and ATRN was investigated in F_2_-generation bulls. It was reported that ASIP is a potential antagonist of the MC1R and MC4R (Lu et al., 1994; Yang et al., 1997) and binds to ATRN with lower affinity (He et al., 2001). The expression of *MC1R* and *MC4R* in bovine SCF corresponds to observations in human (Hoch et al., 2007), but differs from mice, where *Mc1r* is usually expressed in skin and *Mc4r* in brain (Mountjoy et al., 1992; Gantz et al., 1993; Wilson et al., 1999). This may suggest a similar function of MC1R and MC4R in bovine and human. Tissue specific effects of secreted ASIP in cattle are supported by the observation that MC1R was only steadily expressed in SCF, liver, and MLD, whereas the mRNA of MC4R could be stably detected in PF, OF, IF, and liver. Furthermore, the ubiquitous expression of *ATRN* in bovine tissues was firstly reported, in consistence with the expression of *ATRN* in numerous tissues in mice and human (Nagle et al., 1999; Tang et al., 2000).

Liver is an important organ with function in energy metabolism, but also in regulation of adiposity and body weight (Fam et al., 2012). Graphodatskaya et al. (2006) found that *ASIP* was not expressed in bovine liver, whereas Girardot et al. (2005) detected a weak band of *ASIP* mRNA by northern blot. Our results show that *ASIP* mRNA cannot always be detected in liver of HCF or LCF bulls, but was highly expressed in Exon2C bulls due to the L1-BT element. However, the potential receptors were expressed in liver of all three groups of bulls. This indicates that liver may be one of the target organs for ASIP if the protein circulates in low concentrations like some hormones. Further research is necessary to elucidate whether ASIP circulates, with more sensitive methods, and whether it can affect the fat metabolism in bovine liver. Expression differences of ASIP receptors, as shown for *MC4R* and *ATRN* in several tissues, may also be involved. Observed expression differences were not caused by SNPs in the promotor regions of the receptor genes. Sequence analysis of the potential promotor region of *MC4R* demonstrated no difference between HCF and LCF bulls. One coding SNP (c. 1069 C>G) in *MC4R* with known association to back fat thickness, marbling, and carcass weight in cattle (Huang et al., 2010; Liu et al., 2010; Seong et al., 2012) and a novel SNP (c. 1202 G>A) were identified, but allele frequencies were not different between HCF and LCF bulls. Moreover, a new SNP (g. −236 G>C) for *ATRN* located in the potential promotor region was verified. But it still cannot explain the expression difference between HCF and LCF bulls, as there was no obvious relationship between the SNP and carcass fat traits in our population.

The expression level of *ASIP* and *ATRN* showed a positive correlation, whereas *MC4R* exhibited a negative correlation with fat deposition in F_2_-generation bulls of our study. This is in concordance with studies in mice and human. You et al. (2016) reported increased body weight and white adipose tissue mass for the *Mc4r* KO mice. Furthermore, Møller et al. (2015) demonstrated that the agonist of MC4R can lead to a significant stimulation of lipolysis in white adipose tissue of human. Agouti signaling protein has been identified as an antagonist to melanocortin receptors (Willard et al., 1995). When the C-terminus of ASIP binds to MC1R or MC4R, it can lead to an inhibition of cAMP accumulation in the cells (Chai et al., 2003). Furthermore, ATRN can interact with the N-terminus of ASIP, which initiates receptor trafficking to the lysosome and results in the degradation of melanocortin receptors (Overton and Leibel, 2011). Overton and Leibel (2011) demonstrated that knockdown of *Atrn* caused an increased level of MC4R at the cell surface. This suggests that with a certain extracellular concentration of ASIP, a higher cell surface level of ATRN can promote the degradation of melanocortin receptors, which indirectly influences the intracellular cAMP level. It is well known that cAMP plays an important role in lipolysis in adipose tissue and energy metabolism in liver and muscle (Madsen and Kristiansen, 2010; Ravnskjaer et al., 2016). Studies demonstrated elevated cAMP levels in several genetically modified animal models, which led to an increase of lipolysis (Choi et al., 2006; Pagnon et al., 2012). In liver and muscle, the increase of the cAMP level promotes energy metabolism, such as glycogenolysis (Studer and Borle, 1982; Ezrailson et al., 1983). However, it still needs to be further investigated whether the interaction between ASIP and its receptors can exert influence on fat deposition and distribution in cattle through a cAMP dependent or independent mechanism.

## Conclusion

The study confirmed associations between *ASIP* expression and fat deposition in cattle. Overexpression of ASIP in 17 out of 246 F2-generation bulls was paralleled by the abundance of a transposon-derived transcript, named Exon2C. This insertion in the *ASIP* locus led to an ectopic expression of *ASIP* in tissues and cells where it is usually not detectable. However, consequences of the overexpression for protein abundance or fat deposition could not be detected with the applied methods.

In contrast to Exon2C bulls, *ASIP* cannot always be detected in liver, OF, and IF of other bulls, whereas potential receptors like *MC4R* and *ATRN* are expressed in these tissues. This suggests that liver and adipose tissues could be important target organs for ASIP in bovine if the protein is secreted and circulates in low concentrations like some hormones. Receptors for ASIP could have a major function, because the expression levels were significantly different in several tissues between HCF and LCF bulls. In summary, the results indicate that ASIP and ATRN may have a positive effect and MC4R a negative effect on fat deposition in bovine.

## Author contributions

YL performed most experiments and analyses, and drafted the manuscript. EA designed and performed parts of the experiments and analyses, helped to draft the manuscript and revised it. LS performed experiments and revised the manuscript. CK provided and analyzed data, and revised the manuscript. RY and ZZ supervised parts of the work and revised the manuscript. SM designed and supervised the work, revised the manuscript. All authors approved the final version of the manuscript.

### Conflict of interest statement

The authors declare that the research was conducted in the absence of any commercial or financial relationships that could be construed as a potential conflict of interest.

## Abbreviations

α-MSH: α-melanocyte stimulating hormone
ASIP: agouti signaling protein
ATRN: attractin
HCF: high carcass fat
IF: intestinal fat
IMF: intramuscular fat
LCF: low carcass fat
MC1R: melanocortin receptor 1
MC4R: melanocortin receptor 4
MLD: musculus longissimus dorsi
OF: omental fat
PCR: polymerase chain reaction
PF: perirenal fat
qPCR: quantitative polymerase chain reaction
SCF: subcutaneous fat.
