# Supplementary material for: Agouti Signaling Protein and Its Receptors as Potential Molecular Markers for Intramuscular and Body Fat Deposition in Cattle
Source: Front Physiol. 2018 Mar 6;9:172. doi: 10.3389/fphys.2018.00172 (PMC5845533; doi:10.3389/fphys.2018.00172)
Supplement: Supplementary file 1 [file Image1.PDF]

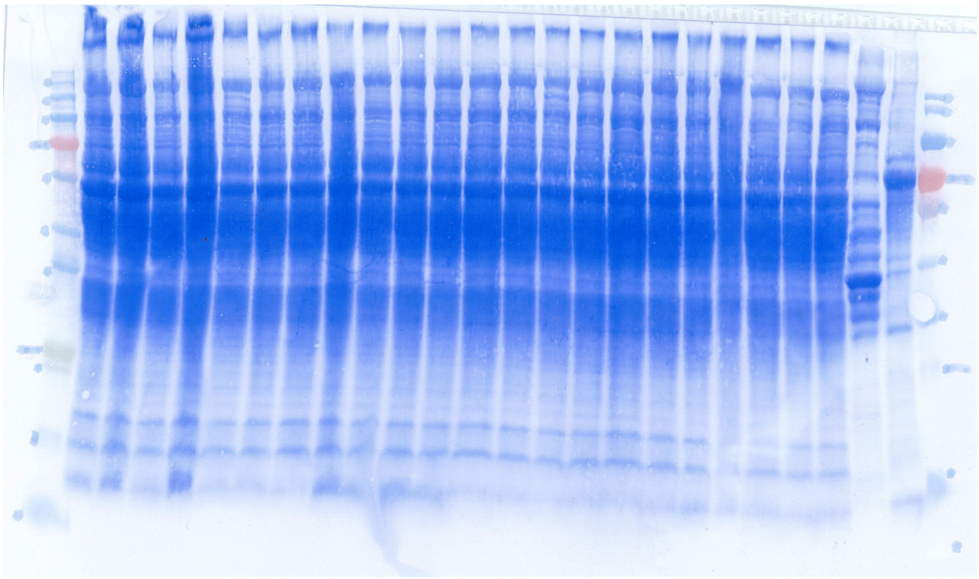

Figure S1: Coomassie stained blot image as used for protein normalization to total protein per lane.
