# Supplementary material for: Agouti Signaling Protein and Its Receptors as Potential Molecular Markers for Intramuscular and Body Fat Deposition in Cattle
Source: Front Physiol. 2018 Mar 6;9:172. doi: 10.3389/fphys.2018.00172 (PMC5845533; doi:10.3389/fphys.2018.00172)
Supplement: Supplementary file 2 [file Image2.PDF]

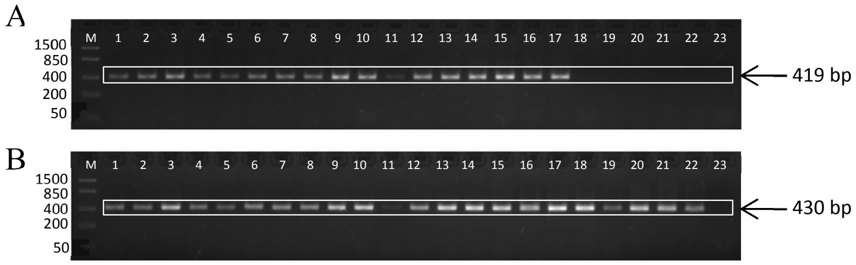

Figure S2: Detection of L1-BT element (Exon2C) at bovine *ASIP* locus in F_2-generation bulls (Charolais × Holstein cross) slaughtered at 18 months of age. Specific PCR products at 419 bp (A) represent the genomic 5'- junctions of L1-BT element. The lengths of amplicons in (B) are 430 bp which spans the genomic region without L1-BT element. Lanes: M – size marker, 1-17: heterozygous Exon2C bulls, 18 and 19: HCF bulls, 20-22: LCF bulls, 23: H_2O.
