# Supplementary material for: Agouti Signaling Protein and Its Receptors as Potential Molecular Markers for Intramuscular and Body Fat Deposition in Cattle
Source: Front Physiol. 2018 Mar 6;9:172. doi: 10.3389/fphys.2018.00172 (PMC5845533; doi:10.3389/fphys.2018.00172)
Supplement: Supplementary file 3 [file Image3.PDF]

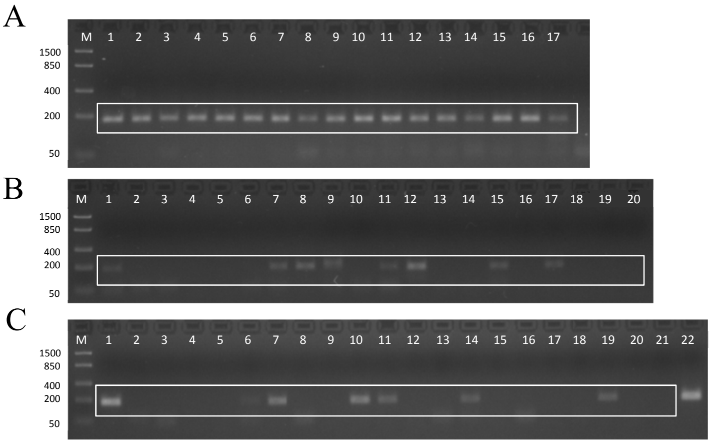

Figure S3: Detection of *ASIP* mRNA expression in liver tissue from F_2-generation bulls (Charolais × Holstein cross) slaughtered at 18 months of age. PCR products with 181 bp length represent the expression of *ASIP* mRNA. Lanes: M – size marker, A: 1-17 Exon2C bulls, B: 1-20 HCF bulls, C: 1-20 LCF bulls, 21 H_2O, 22 positive control. HCF – high carcass fat, LCF – low carcass fat.
