# Supplementary material for: Agouti Signaling Protein and Its Receptors as Potential Molecular Markers for Intramuscular and Body Fat Deposition in Cattle
Source: Front Physiol. 2018 Mar 6;9:172. doi: 10.3389/fphys.2018.00172 (PMC5845533; doi:10.3389/fphys.2018.00172)
Supplement: Supplementary file 4 [file Image4.PDF]

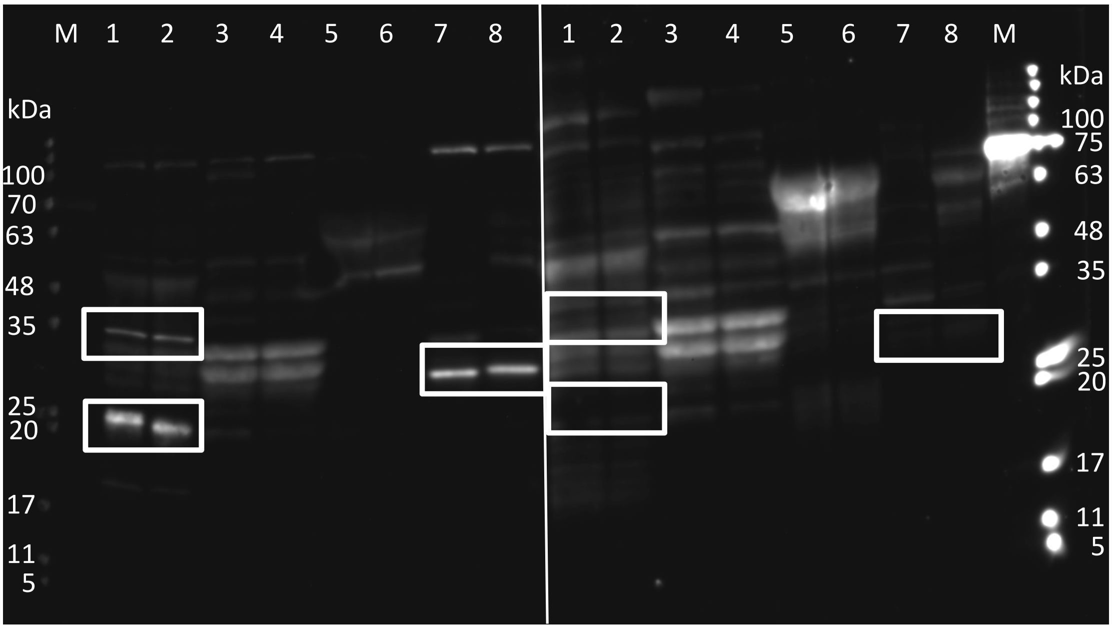

Figure S4: Western blot of MC4R in liver, muscle and subcutaneous adipose tissue of F_2-generation bulls (Charolais × Holstein cross) slaughtered at 18 months of age. The right panel was incubated with antibody previously blocked with the antigen peptide to determine unspecific bindings. The white boxes indicate specific, blockable bands in bovine liver and mouse muscle and adipose tissue. Lanes: M – size marker, 1-2 bovine liver, 3-4 bovine muscle, 5-6 bovine subcutaneous fat, 7 murine muscle, 8 murine subcutaneous fat.
