# Supplementary material for: Agouti Signaling Protein and Its Receptors as Potential Molecular Markers for Intramuscular and Body Fat Deposition in Cattle
Source: Front Physiol. 2018 Mar 6;9:172. doi: 10.3389/fphys.2018.00172 (PMC5845533; doi:10.3389/fphys.2018.00172)
Supplement: Supplementary file 5 [file Table1.PDF]

Supplemental Material

Table S1: Selected phenotypic traits of 3 groups of F_2-generation bulls (Charolais × Holstein cross) slaughtered at 18 months of age

	Group						
	Exon2C		HCF		LCF		P -
Trait	LSmean	SE	LSmean	SE	LSmean	SE	value
Age, days	547	1	547	1	547	1	0.8886
Body weight, kg	701[a]	13	712[a]	11	626[b]	16	<.0001
Average daily gain, kg	0.69[a]	0.01	0.70[a]	0.01	0.61[b]	0.02	<.0001
Cold carcass weight, kg	397.6[a]	7.3	402.8[a]	6.7	358.5[b]	10.1	0.0005
Liver, kg	7.55[a]	0.21	7.87[a]	0.19	6.30[b]	0.24	<.0001
Meat, %	76.96[a]	0.25	75.58[b]	0.31	77.68[a]	0.38	<.0001
Bones, %	14.37[b]	0.24	13.74[b]	0.13	16.09[a]	0.40	<.0001
Tendons, %	2.38[b]	0.06	2.43[b]	0.05	2.72[a]	0.07	0.0008
Carcass fat, kg	68.61[b]	3.18	95.47[a]	1.38	35.88[c]	1.38	<.0001
Carcass fat, %	17.19[b]	0.76	23.52[a]	0.46	10.04[c]	0.27	<.0001
Carcass protein, %	14.35[b]	0.15	13.21[c]	0.12	15.44[a]	0.12	<.0001
Subcutaneous fat, %	6.29[b]	0.26	8.25[a]	0.28	3.44[c]	0.14	<.0001
Intestinal fat, %	1.16[b]	0.11	1.74[a]	0.10	0.98[b]	0.07	<.0001
Omental fat, %	2.32[b]	0.16	2.85[a]	0.13	1.73[c]	0.10	<.0001
Perirenal fat, %	2.63[a]	0.17	2.65[a]	0.11	1.62[b]	0.10	<.0001
M. longissimus weight, kg	8.26	0.17	7.85	0.19	7.80	0.28	0.3161
IMF[1] of M. longissimus, %	3.82[b]	0.39	5.61[a]	0.44	1.53[c]	0.09	<.0001
Marbling fleck area percentage, %	7.00[a]	0.63	8.01[a]	0.59	2.31[b]	0.24	<.0001
Number of marbling flecks	585[b]	33	756[a]	40	366[c]	43	<.0001
Distance between marbling flecks, mm	1.85[b]	0.04	1.71[b]	0.03	2.23[a]	0.17	0.0017

[a-c] means without a common superscript differ ($P < 0.05$).
[1]IMF – intramuscular fat content
